# Histological variant as the significant predictor of survival in patients with lymph node positive urothelial carcinoma of the bladder

**DOI:** 10.1038/srep09626

**Published:** 2015-05-11

**Authors:** Hyung Suk Kim, Kyung Chul Moon, Chang Wook Jeong, Cheol Kwak, Hyeon Hoe Kim, Ja Hyeon Ku

**Affiliations:** 1 Department of Urology, Seoul National University College of Medicine, Seoul, Korea; 2 Department of Pathology, Seoul National University College of Medicine, Seoul, Korea

## Abstract

The aim of this study was to evaluate the impact of histological variants of urothelial carcinoma (UC) on survival outcomes in patients with lymph node (LN) positive UC of the bladder. We reviewed and analyzed the clinical data from 424 patients who underwent radical cystectomy (RC) with pelvic lymph node dissection (PLND) for UC of the bladder and who did not receive neoadjuvant chemotherapy in our institution between 1991 and 2012. In total, 92 patients (21.7%) had histologically confirmed LN positive disease. In the LN negative group (332 patients), histological variants of UC were not a significant predictor in univariate analysis. However, in the LN positive group, histological variants of UC were a significant independent prognostic factor of overall survival (hazard ratio (HR) 3.54; 95% confidence interval (CI) 1.77–7.08, *p* < 0.001) and cancer specific survival (HR 3.66; 95% CI 1.69–7.90, *p* = 0.001) in both uni-variate and multivariate Cox regression analyses. The presence of histological variants of UC may indicate a worse prognosis in LN positive patients after RC with PLND for UC of the bladder and more aggressive adjuvant therapy may be required for the improvement of postoperative survival.

Urothelial carcinoma (UC) of the bladder has become a significant health problem worldwide, with estimates of 74,690 new cases and 15,580 deaths caused by bladder cancer in the United States in 2014^1^. It is also estimated that in Korea, a total of 4,173 cases were newly diagnosed and 1,264 deaths were attributed to bladder cancer in 2014^2^. Approximately 25–30% of patients with UC of the bladder show muscle invasive disease at initial diagnosis, for which the standard care treatment is radical cystectomy (RC) with pelvic lymph node dissection (PLND), which can also be applied to high-risk non-muscle invasive disease^3^,^4^,^5^. It has been demonstrated in several studies that approximately 25% of patients with T1-4N0M0 bladder cancer who receive RC with PLND present with lymph node (LN) positive disease^4^,^6^,^7^,^8^. Furthermore, LN involvement in bladder cancer has been known as an independent adverse prognostic factor^6^,^7^,^9^.

Many variables, such as primary T stage, lymphovascular invasion (LVI), LN profiles (total number of removed LNs, total number of positive LNs, and lymph node density (LND)), have been suggested as significant factors that are associated with the survival outcome of patients with positive LNs^8^,^10^,^11^,^12^,^13^,^14^,^15^,^16^. Recent studies of, the histological variants of UC, which account for less than 10% of all bladder cancer cases, have been reported^17^,^18^,^19^,^20^. Histological variants of UC are generally known to result in an unfavorable prognosis in association with high grade and advanced pathologic stage at the time of presentation. Histological variants of UC may also drive lymphatic spread, or conversely, they may be a marker for locally advanced disease^20^. However, few studies have evaluated the prognostic value of histological variants of UC in node positive bladder cancer due to the paucity of such cases.

In the present study, we aim to assess the significant prognostic factors correlated with survival outcomes in LN positive patients undergoing RC with PLND for UC of the bladder, particularly focusing on the prognostic significance of histological variants of UC.

## Results

Table 1 presents the clinical and pathological characteristics of all patients (n = 424) and comparative analysis results between the LN negative group (n = 332) and the LN positive group (n = 92). Patients with positive LNs showed a higher frequency of more advanced disease (pT2 or pT3/T4), high grade, LVI, positive perivesical margin, and extended PLND in comparison with LN negative patients. In the entire population and in each subgroup, the majority of the histological variants of UC displayed features of squamous differentiation (Supplemental Table 1). On multivariate logistic regression analysis, the significant risk factors associated with LN involvement after RC and PLND were more advanced disease (pT2 or pT3/T4), LVI, and positive perivesical margins (Supplemental Table 2).

**Figure 1:**
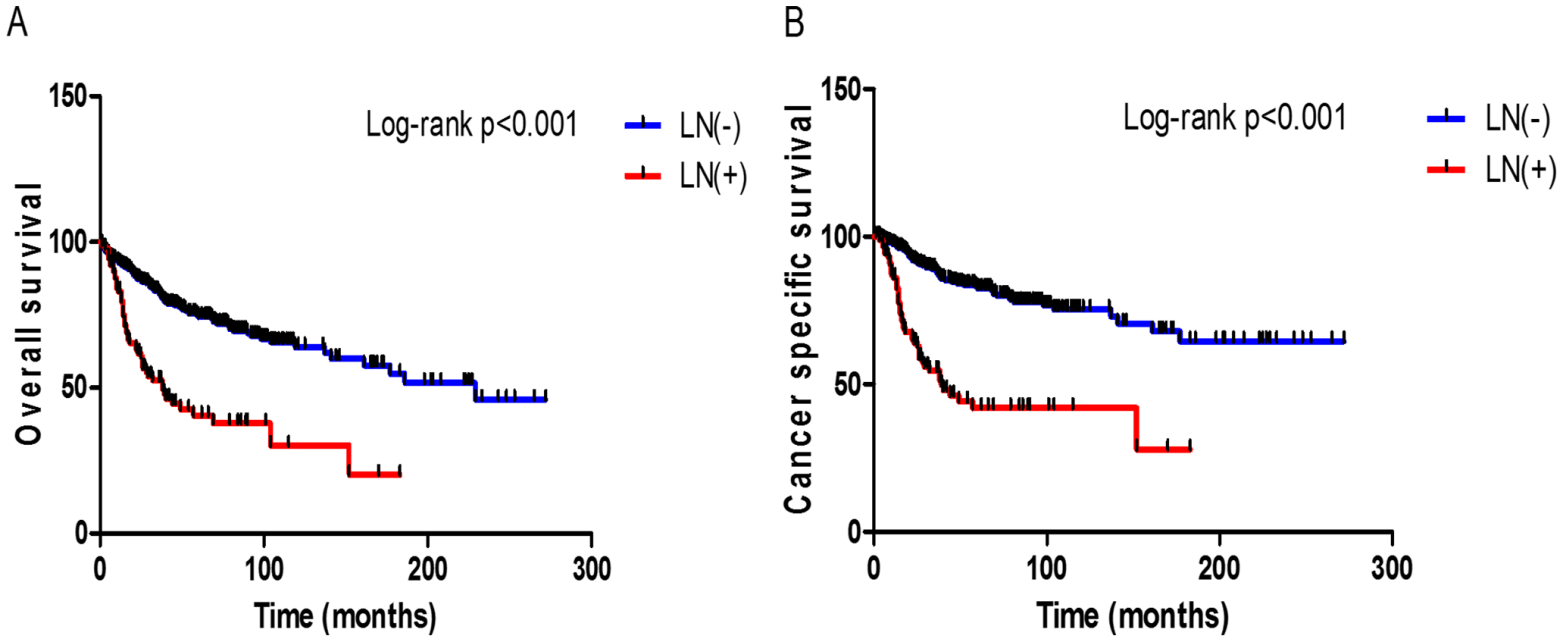
Kaplan-Meier curves for overall survival (A) and cancer specific survival (B) according to the presence of lymph node (LN) involvement after radical cystectomy with pelvic lymph node dissection.

In this study cohort, there were 142 (33.5%) patients who died of any cause with a median follow-up duration of 40 months (interquartile range (IQR): 23–85 months) and 104 of these deaths (24.5%) were attributed to UC of the bladder. The median follow-up duration was 48 months (IQR: 25–90 months) for the LN negative group and 26 months (IQR: 13–48.2 months) for the LN positive group (*p* < 0.001). The three- and five-year OS rates were 82.1% and 74.4%, respectively, for the LN negative group versus 52.0% and 40.0% for the LN positive group (*p* < 0.001, log-rank test) (Figure 1A). CSS rates for the same time periods were 87.9% and 82.5%, respectively, for the LN negative group versus 54.5% and 41.9% for the LN positive group (*p* < 0.001, log-rank test) (Figure 1B).

**Figure 2:**
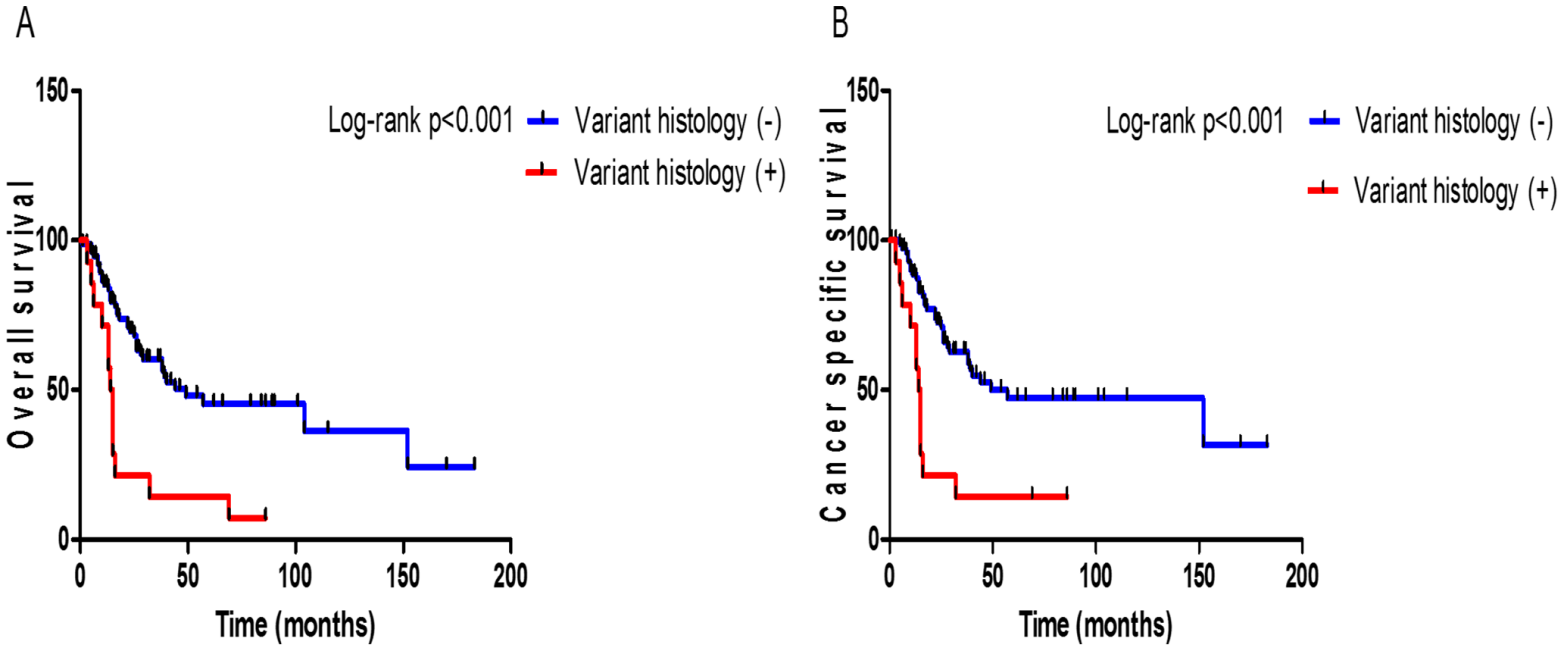
Kaplan-Meier curves for overall survival (A) and cancer specific survival (B) according to the presence of histological variant of urothelial carcinoma (UC) in lymph node positive group.

In LN positive group, patients with variant histology of UC showed a significantly reduced 3-year OS rate than conventional UC patients (13% vs. 56%, respectively; *p* < 0.001; Fig. 2A). Also, there was significant difference in the 3-year CSS rate between variant histology of UC and conventional UC patients (14% vs. 59%, respectively; *p* < 0.001; Fig. 2B).

To assess the factors related to survival in the LN positive group after surgery, univariate and multivariate analyses using the Cox proportional hazard model were performed. As a result of multivariate analysis adjusting for the significant variables identified in the univariate analysis, histological variants of UC (*p* < 0.001), LVI (*p* = 0.003), and dichotomized LND of more than 18% (*p* = 0.013) remained as the significant predictors of a shorter OS (Table 2). Similarly, histological variants of UC (*p* = 0.001), LVI (*p* = 0.004), a decreased number of removed LNs (*p* = 0.006), and an increased number of positive LNs (*p* = 0.001) were identified as the independent prognostic factors of a poorer CSS (Table 3). While variant histology of UC was an independent predictor of OS and CSS in both uni-and multivariate analysis, more advanced disease (pT2 or pT3/T4) and higher nodal stage (pN2/N3) showed no significant correlation with survival outcomes as a result of statistical analyses. (Table 2 and 3).

We also evaluated the variables associated with survival outcomes in the LN negative group. On multivariate analysis, age (*p* = 0.003), BMI (*p* = 0.007), extravesical disease (pT3/T4) and the number of removed LNs (*p* < 0.001) were significant predictors of OS (Supplemental Table 3). Likewise, BMI (*p* = 0.045), LVI (*p* = 0.039), and the number of removed LNs (*p* < 0.001) were independent prognostic factors of CSS on multivariate analysis (Supplement Table 4). Variant histology of UC was not a significant prognostic factor related to OS and CSS in univariate analysis (Supplement Table 3 and 4).

## Discussion

The postoperative prognosis of patients treated with RC and PLND for UC of the bladder can be affected by a variety of clinical and pathological factors^22^,^23^. According to the SWOG trial^24^, neoadjuvant chemotherapy (NACH) is also significant factor associated with improved survival. However, the cases treated with NACH were excluded in the current study cohort, given that NACH may affect the clinical course of bladder cancer and alter the disease progression state, including LN involvement. In addition, for homogeneity of the study cohort, the histological type of bladder cancer involved was confined to the cases showing UC histology in the final pathologic examination of their RC specimen, and non-UCs (squamous cell carcinoma, adenocarcinoma, etc.) were excluded.

In general, the presence of LN involvement is considered to be predictive of a poor survival outcome in patients undergoing RC^6^,^7^,^25^. In this study, the three year CSS rate in the LN positive group was significantly lower than that in the LN negative group (54.5% versus 87.9%, *p* < 0.001) and the definitive variables associated with LN involvement were more advanced disease (pT2 or pT3/T4), LVI, and positive perivesical margin. Likewise, Adel-Latif et al. reported that pT stage, tumor grade, and LVI were independent factors promoting the incidence of nodal involvement and three-year disease-free survival rates were 78.3% and 37.8% in node negative and positive cases, respectively (*p* < 0.001)^6^. The survival results of LN positive patients in our study were slightly higher than those of previously reported studies^4^,^6^,^7^. These findings may result from the survival benefit gained by the higher frequency of adjuvant chemotherapy use (67.4% versus 15.4%, *p* < 0.001) after surgery in LN positive patients.

Several studies demonstrated that although nodal stage N0 is assigned to patients preoperatively, approximately 25% of those may show positive LNs following RC and PLND^6^,^7^,^25^. Therefore, there have been a number of studies dealing with the role of PLND and lymphadenectomy related variables (namely, LN profiles), including the extent of PLND, total number of removed LNs, total number of positive LNs, and the LND at the time of RC. As for the extent of PLND, Dhar et al. demonstrated that compared with limited PLND, extended PLND allows for more accurate staging and improved survival of patients with non-organ confined and LN positive disease^3^. Herr et al. reported that in cases with at nine or more LNs retrieved, improved survival outcomes were obtained in patients with muscle invasive bladder cancer^26^. Several studies have demonstrated that the absolute number of positive LNs affects patient outcomes and survival in patients with node positive bladder cancer^4^,^6^,^7^. However, there is no unified criterion with regard to the cut-off value of removed LNs and positive LNs that is associated with a survival benefit. Recently, the concept of LND has been suggested as a significant predictor of survival in node positive bladder cancer^14^,^15^,^16^.

In the current study, PLND and the LN profiles mentioned above showed a clinical implication in association with survival outcomes at various levels. Even though extended PLND showed a significantly higher performance frequency in LN positive patients compared to patients with negative LNs (26.1% versus 15.7%, *p* = 0.009), it had no prognostic significance with regard to survival outcomes in LN positive or LN negative patients. Since our study cohort consisted of patients who underwent RC with PLND performed by several surgeons, there were individual differences in the decision-making criteria for the extent of PLND to be performed and in the level of expertise in performing PLND, which may contribute to the variation in the quantitative and qualitative aspects of dissected LNs affecting the postoperative prognosis. However, the total number of removed LNs was a significant prognostic factor correlated with OS and CSS (*p* < 0.001 for both OS and CSS) in LN negative patients as well as with CSS (*p* = 0.006) in LN positive patients, suggesting that regardless of the postoperative LN status, assuring an adequate number of LNs are removed during RC is important for improving postoperative survival. This result is also supported by the preceding literature^8^,^21^,^26^. The number of positive LNs (HR 1.22; 95% CI 1.08–1.38, *p* = 0.001) was an independent prognostic factor of CSS, which corresponds to the findings in preceding studies^4^,^6^,^7^. Similar to previous studies^14^,^15^,^16^ using a dichotomized LND variable, we used the median value of 18% as the criterion to dichotomize LND values so that LND (HR 2.09; 95% CI 1.16–3.77, *p* = 0.013) showed a significant prognostic association with OS in LN positive patients.

Other than LN profiles, clinicopathological factors, including age, pathological T stage, LVI, tumor grade, surgical margin status, and NACH have been suggested as significant predictors of survival outcomes in patients treated with RC and PLND^10^,^22^,^23^,^24^. Of these, LVI is known as a well-established independent predictor of clinical and oncological outcomes after RC observed in a variety of studies^11^,^12^,^13^. These studies mainly focused on the prognostic value of LVI in only LN negative UC. In contrast, our result is noteworthy in that LVI was an independent prognostic predictor of OS (HR 2.78; 95% CI 1.41–5.47, *p* = 0.003) and CSS (HR 3.08; 95% CI 1.44–6.58, *p* = 0.004) in patients with LN positive UC of the bladder as well as CSS in LN negative patients. In addition, it was revealed in our study that LVI is a significant risk factor correlated with an increased probability of LN involvement. Therefore, it is possible to assume that LVI as a risk factor for LN involvement has a negative impact on survival outcomes in LN positive patients.

Recently, many studies of the histological variants of UC have been conducted. Although such cases are very rare, they are generally regarded to show poor prognostic results, such as advanced stage, higher grade and LN metastasis^17^,^18^,^19^,^20^. Due to these aggressive clinical features, RC has been recommended as the mainstay therapeutic modality^18^,^19^. However, there have been a few studies concerning the prognostic value of histologic variants of UC in patients presenting positive LNs following RC. In the present study, histologic variants of UC are an independent prognostic predictor of OS (HR 3.54; 95% CI 1.77–7.08, *p* < 0.001) and CSS (HR 3.66; 95% CI 1.69–7.90, *p* = 0.001) exclusively in LN positive patients. The limitations of this analysis were that, although the impact of variant histology on clinical outcomes may vary depending on the subtype, we considered all subtypes as one entity during the analytic process of our study cohort due to the low number of cases (15 patients). Moreover, we included the cases of histological variants found in TURB tumor specimens as well as specimens obtained from RC. Wasco et al. reported that histologic variants of UC observed in TURB tumor specimens were an independent predictor of extravesical disease, but they were not a significant predictor of disease specific survival^27^. However, in our study, histological variants in the LN positive group showed a significant correlation with more advanced pathologic T stage (all more than pT2, *p* = 0.015), higher overall mortality (*p* = 0.001) and cancer specific mortality (*p* = 0.008) than pure UC, which may ultimately lead to worse survival outcomes for LN positive patients.

In conclusion, the existing significant factors related to survival outcomes in patients treated by RC and PLND for bladder cancer were also identified in this study. Above all, the results of our study confirm the prognostic value of histological variants of UC as a predictor of survival outcomes exclusively in LN positive patients. This finding should be importantly considered when determining further treatment plan in LN positive bladder cancer patients. In other words, the additional aggressive adjuvant treatments, such as chemotherapy or radiotherapy, may be required to improve postoperative survival in patients with positive LNs after RC presenting with a variant histology of UC. However, since this study was based on the retrospective design, our results should be verified in a prospective study.

## Methods

### Ethics statement

This study design and the use of patients' information stored in the hospital database were approved by the Institutional Review Board (IRB) at the Seoul National University Hospital (SNUH). The approval number is H-1407-196-600. We were given exemption from getting informed consents by the IRB because the present study is a retrospective study and personal identifiers were completely removed and the data were analyzed anonymously. Our study was conducted according to the ethical standards laid down in the 1964 Declaration of Helsinki and its later amendments.

### Study population

After obtaining approval from the IRB of SNUH, we retrospectively reviewed the electronic medical charts of 487 patients who underwent RC with PLND for bladder cancer at our institution from January 1991 to December 2012. Patients who had a diagnosis of non-urothelial carcinoma (n = 13) following analysis of their final RC specimen and those with a history of treatment with neoadjuvant chemotherapy (NACH) (n = 50) were excluded from the study. Consequently, a total of 424 patients were available for our final analysis.

### Data Acquisition

RC and PLND were carried out as previously described in detail^21^. The indications for RC included patients with muscle-invasive carcinoma of the bladder and recurrent T1 disease or *carcinoma in situ* (CIS) that had been unresponsive to intravesical chemotherapy. The evaluation of pathological specimens of RC and dissected LNs was conducted by a staff pathologist with genitourinary expertise. Pathological staging and grading were assigned according to the 2010 Tumor-Node-Metastasis classification of 7th American Joint Committee on Cancer (AJCC) and the 2004 World Health Organization (WHO) system, respectively. Therefore, we re-evaluated the pathologic stage and grade of patients who underwent surgery prior to application of updated guideline systems. To facilitate statistical analyses, T staging was categorized into non-muscle invasive (pT0/Ta/T1/CIS), muscle invasive (pT2) and extravesical disease (pT3/T4). Histological variants of UC included those found during transurethral resection of the bladder (TURB) prior to RC, those in the final RC specimen or both. LVI was defined as the presence of tumor cells within an endothelium-lined space without underlying muscular walls. Other analyzed covariates, including age, gender, body mass index (BMI), American Society of Anesthesiologists (ASA) score, CIS, perivesical margin, total number of removed LNs, total number of positive LNs, LND, and adjuvant chemotherapy were obtained from patient records. LND was defined as the ratio of positive LNs to the total number of removed LNs^14^,^15^. The duration of survival was calculated from the date of surgery to the date of last follow-up or death. Patients who were alive, with or without disease, were censored from the relevant analyses. Cause of death was determined by the responsible physicians and death certificates.

### Statistical analyses

Clinical and pathological characteristics were compared between LN negative and positive groups using chi-squared or Fisher's exact tests for categorical variables and Student's *t*-test for continuous variables. Survival outcomes measured as overall survival (OS) and cancer specific survival (CSS) were calculated using the Kaplan-Meier method and compared using a log-rank test. To assess the factors associated with survival in each subgroup, univariate analyses using the Cox proportional hazards model were conducted and significant variables identified in the univariate analyses were finally entered into a multivariate Cox regression analysis to evaluate the definitive predictors. All statistical analyses were carried out using the SPSS software version 18.0 (SPSS Inc., Chicago, IL, USA) and two-sided *p*-values of less than 0.05 were considered to be statistically significant.

## Supplementary Material

Supplementary Information Supplementary information

## Figures and Tables

**Table 1:** Clinico-pathological parameters of the study cohort and comparative analysis results of parameters between lymph node negative and positive group

	Overall (n = 424)	LN(-) (n = 332)	LN(+) (n = 92)	p-value
Age (year), mean ± SD	63.9 ± 10.2	63.8 ± 10.2	64.2 ± 10.2	0.700
<60 yrs	125(29.5%)	97(29.2%)	28(30.4%)	0.821
≥60 yrs	299(70.5%)	235(70.8%)	64(69.6%)	
Gender, n (%)				
Male	367(86.6%)	288(86.7%)	79(85.9%)	0.827
Female	57(13.4%)	44(13.3%)	13(14.1%)	
BMI (kg/m2), mean ± SD	23.3 ± 3.7	23.3 ± 2.9	23.4 ± 5.8	0.777
<25	305(71.9%)	238(72.8%)	67(73.6%)	0.873
≥25	113(26.7%)	89(27.2%)	24(26.4%)	
ASA score, n(%)				
0	3(0.7%)	2(0.6%)	1(1.1%)	0.328
1	171(40.3%)	132(40.5%)	39(43.8%)	
2	219(51.7%)	174(53.4%)	45(50.6%)	
3	21(5.0%)	18(5.5%)	3(3.4%)	
4	1(0.2%)	0(0%)	1(1.1%)	
Pathologic T stage, n (%)				
Non-muscle invasive (pT0/Ta/T1/CIS)	184(43.4%)	174(52.4%)	10(10.9%)	<0.001
Muscle invasive (pT2)	87(20.5%)	66(19.9%)	21(22.8%)	
Extravesical (pT3/T4)	153(36.1%)	92(22.7%)	61(66.3%)	
Pathologic grade, n(%)				
Low grade	70(16.5%)	65(19.6%)	5(5.4%)	0.001
High grade	353(83.3%)	266(80.4%)	87(94.6%)	
Carcinoma in situ, n (%)				
Absent	303(71.5%)	232(69.9%)	71(77.2%)	0.170
Present	121(28.5%)	100(30.1%)	51(22.8%)	
Lymphovascular invasion, n (%)				
Absent	279(65.8%)	249(75.0%)	30(32.6%)	<0.001
Present	145(34.2%)	83(25.0%)	62(67.4%)	
Perivesical margin, n (%)				
Absent	414(97.6%)	330(99.4%)	84(95.2%)	<0.001
Present	10(2.4%)	2(0.6%)	8(4.8%)	
Variant histology of UC, n (%)				
Absent	367(86.6%)	290(87.3%)	77(83.7%)	0.363
Present	57(13.4%)	42(12.7%)	15(16.3%)	
Extent of PLND, n (%)				
Limited	62(14.4%)	55(16.6%)	6(6.5%)	0.009
Standard	286(67.5%)	224(67.7%)	62(67.4%)	
Extended	76(17.9%)	52(15.7%)	24(26.1%)	
Lymph node status, n (%)				
N1			37(40.2%)	
N2/N3			55(59.8%)	
Mean total number of removed LNs ± SD (median, IQR)	14.8 ± 9.9 (14, 8–20)	14.3 ± 9.2 (13, 7–20)	16.6 ± 12.0 (14.5, 8–21.75)	0.093
Mean total number of positive LNs ± SD (median, IQR)			3.2 ± 3.0 (2, 1–4)	
Mean % LND ± SD (median, IQR)			29.3 ± 27.4 (17.8, 8.3–43.2)	
LND, dichotomized, n (%)				
≤18			46(50.0%)	
>18			46(50.0%)	
Adjuvant chemotherapy, n (%)				
Not done	311(73.3%)	281(84.6%)	30(32.6%)	<0.001
Done	113(26.7%)	51(15.4%)	62(67.4%)	
Mean OS f/u duration ± SD, n (%) (median, IQR)	59.1 ± 52.9 (40, 23–85)	65.1 ± 55.2 (48, 25–90)	37.5 ± 36.5 (26, 13–48.25)	<0.001
Alive	282(66.5%)	241(72.6%)	41(44.6%)	<0.001
Death	142(33.5%)	91(27.4%)	51(55.4%)	
Mean CSS f/u duration ± SD, n (%) (median, IQR)	59.1 ± 52.9 (40, 23–85)	65.1 ± 55.1 (48, 25–90)	37.5 ± 36.5 (26, 13–48.25)	<0.001
Alive	320(75.5%)	273(82.2%)	47(51.1%)	<0.001
Death	104(24.5%)	59(17.8%)	45(48.9%)	

BMI = body mass index; ASA = American Society of Anesthesiologists; UC = urothelial carcinoma; PLND = pelvic lymph node dissection; LN = lymph node; LND = lymph node density; OS = overall survival; CSS = cancer specific survival; SD = standard deviation; IQR = interquartile range.

**Table 2:** Uni-variate and multivariate Cox regression analyses results for evaluating variables associated with overall survival in lymph node positive group

	Univariate analysis			Multivariate analysis		
	Unadjusted HR	95% CI	p-value	Adjusted HR	95% CI	p-value
Pathologic T stage						
Non-muscle invasive (pT0/Ta/T1/CIS)	Reference			Reference		
Muscle invasive (pT2)	1.08	0.32–3.59	0.898	0.87	0.22–3.48	0.841
Extravesical (pT3/T4)	3.18	1.10–9.13	0.032	1.23	0.33–4.58	0.753
Variant histology of UC						
Absent	Reference			Reference		
Present	3.53	1.88–6.64	<0.001	3.54	1.77–7.08	<0.001
CIS						
Absent	Reference			Reference		
Present	0.42	0.18–0.94	0.035	0.48	0.20–1.12	0.093
LVI						
Absent	Reference			Reference		
Present	2.05	1.09–3.88	0.026	2.78	1.41–5.47	0.003
Pathologic nodal stage						
N1	Reference					
N2	1.70	0.94–3.08	0.080			
N3	1.78	0.59–5.34	0.305			
Number of removed LNs (continuous)	0.96	0.93–0.99	0.022	0.98	0.95–1.01	0.335
LND, dichotomized						
<18%	Reference			Reference		
≥18%	2.27	1.28–4.02	0.005	2.09	1.16–3.77	0.013

HR = hazard ratio; CI = confidence interval; UC = urothelial carcinoma; CIS = carcinoma in situ; LVI = lymphovascular invasion; LND = lymph node density.

**Table 3:** Uni-variate and multivariate Cox regression analyses results for evaluating variables associated with cancer specific survival in lymph node positive group

	Univariate analysis			Multivariate analysis		
	Unadjusted HR	95% CI	p-value	Adjusted HR	95% CI	p-value
Pathologic T stage						
Non-muscle invasive (pT0/Ta/T1/CIS)	Reference			Reference		
Muscle invasive (pT2)	1.06	0.27–4.25	0.932	0.69	0.14–3.43	0.655
Extravesical (pT3/T4)	3.66	1.12–11.97	0.032	1.34	0.31–5.91	0.695
Variant histology of UC						
Absent	Reference			Reference		
Present	3.48	1.77–6.85	<0.001	3.66	1.69–7.90	0.001
LVI						
Absent	Reference			Reference		
Present	2.47	1.22–5.01	0.012	3.08	1.44–6.58	0.004
Pathologic nodal stage						
N1	Reference					
N2	1.80	0.95–3.42	0.070			
N3	2.14	0.70–6.55	0.181			
Number of removed LNs (continuous)	0.96	0.93–0.99	0.023	0.94	0.90–0.98	0.006
Number of positive LNs (continuous)	1.11	1.01–1.22	0.022	1.22	1.08–1.38	0.001
LND, dichotomized						
<18%	Reference			Reference		
≥18%	2.67	1.44–4.95	0.002	1.08	0.40–2.90	0.879

HR = hazard ratio; CI = confidence interval; UC = urothelial carcinoma; LVI = lymphovascular invasion; LND = lymph node density.
