# Supplementary material for: Histological variant as the significant predictor of survival in patients with lymph node positive urothelial carcinoma of the bladder
Source: Sci Rep. 2015 May 11;5:9626. doi: 10.1038/srep09626 (PMC4427059; doi:10.1038/srep09626)
Supplement: Supplementary Information [file srep09626-s1.pdf]

TITLE

Histological variant as the significant predictor of survival in patients with lymph node positive urothelial carcinoma of the bladder

SHORT TITLE

The prognostic value of histological variant in node positive urothelial bladder cancer

AUTHORS AND INSTITUTIONS

Hyung Suk Kim[1], Kyung Chul Moon[2], Chang Wook Jeong[1], Cheol Kwak[1], Hyeon Hoe Kim[1], Ja Hyeon Ku[1★]

[1]Department of Urology, Seoul National University College of Medicine, Seoul, Korea

[2]Department of Pathology, Seoul National University College of Medicine, Seoul, Korea

★ CORRESPONDENCE

Ja Hyeon Ku, M.D. PhD.

Department of Urology, Seoul National University Hospital,

101 Daehak-ro, Jongno-gu, Seoul 110-744, Korea

Tel: 82-2-2072-0361

Fax: 82-2-742-4665

E-mail: kuuro70@snu.ac.kr

Supplemental Table 1. Distributions of histological variants of UC

Histological variants of UC	Overall (n=57)	LN(-) (n=42)	LN(+) (n=15)
Types			
Squamous differentiation, n (%)	34 (60%)	27 (64%)	7 (47%)
Glandular differentiation, n (%)	6 (10%)	4 (9.6%)	2 (13%)
Nested variant, n (%)	2 (3.5%)	0 (0%)	2 (13%)
Clear cell, n (%)	1 (1.8%)	1 (2.4%)	0 (0%)
Plasmacytoid, n (%)	4 (7%)	2 (4.8%)	2 (13%)
Intracytoplasmic mucin production, n (%)	1 (1.8%)	0 (0%)	1 (7%)
Signet ring cell, n (%)	1 (1.8%)	1 (2.4%)	0 (0%)
Micropapillary, n (%)	3 (5.3%)	2 (4.8%)	1 (7%)
Sarcomatoid, n (%)	5 (8.8%)	5 (12%)	0 (0%)
Pathologic T stage, n (%)			
Non-muscle invasive (pT0/Ta/T1/CIS)	11 (19.3%)	11 (26.2%)	0 (0%)
Muscle invasive (pT2)	13 (22.8%)	12 (28.6%)	1 (6.7%)
Extravesical (pT3/T4)	33 (57.9%)	19 (45.2%)	14 (93.3%)

UC=urothelial carcinoma; LN=lymph node

Supplemental Table 2. Uni-variate and multivariate logistic regression analyses results for **t**he risk factors associated with lymph node involvement after radical cystectomy and pelvic lymph node dissection

	Univariate analysis			Multivariate analysis		
	Unadjusted OR	95% CI	p-value	Adjusted OR	95% CI	p-value
Pathologic T stage						
Non-muscle invasive (pT0/Ta/T1/CIS)	Reference			Reference		
Muscle invasive (pT2)	5.54	2.48-12.38	<0.001	3.82	1.65-8.88	0.002
Extravesical (pT3/T4)	11.53	5.64-23.58	<0.001	5.84	2.62-13.00	<0.001
LVI						
Absent	Reference			Reference		
Present	6.20	3.75-10.24	<0.001	2.66	1.49-4.75	0.001
Pathologic grade						
Low grade	Reference			Reference		
High grade	4.25	1.65-10.89	0.003	0.99	0.33-2.96	0.990
Perivesical margin						
Absent	Reference			Reference		
Present	15.7	3.27-75.37	0.001	5.11	1.04-25.14	0.045

OR=odds ratio; CI=confidence interval; LVI=lymphovascular invasion

Supplemental Table 3. Uni-variate and multivariate Cox regression analyses results for evaluating **v**ariables associated with overall survival in lymph node negative group

	Univariate analysis			Multivariate analysis		
	Unadjusted HR	95% CI	p-value	Adjusted HR	95% CI	p-value
Age, year,						
< 60yrs	Reference			Reference		
≥ 60yrs	2.17	1.30-3.64	0.003	2.25	1.33-3.82	0.003
BMI(kg/m2),						
<25	Reference			Reference		
≥25	0.43	0.24-0.75	0.003	0.47	0.27-0.81	0.007
Pathologic T stage						
Non-muscle invasive (pT0/Ta/T1/CIS)	Reference			Reference		
Muscle invasive (pT2)	0.69	0.35-1.37	0.291	0.53	0.25-1.10	0.089
Extravesical (pT3/T4)	2.92	1.87-4.55	<0.001	2.39	1.40-4.07	0.001
Variant histology of UC						
Absent	Reference					
Present	1.02	0.57-1.80	0.960			
LVI						
Absent	Reference			Reference		
Present	1.99	1.30-3.03	0.001	1.58	0.95-2.65	0.078
Perivesical margin						
Absent	Reference			Reference		
Present	11.26	2.73-46.4	0.001	3.41	0.79-14.68	0.099
Number of LNs removed (continuous)	0.94	0.91-0.97	<0.001	0.94	0.91-0.97	<0.001

HR=hazard ratio; CI=confidence interval; BMI=body mass index; LVI=lymphovascular invasion; LNs=lymph nodes

Supplemental Table 4. Uni-variate and multivariate Cox regression analyses results for evaluating **v**ariables associated with cancer specific survival in lymph node negative group

	Univariate analysis			Multivariate analysis		
	Unadjusted HR	95% CI	p-value	Adjusted HR	95% CI	p-value
BMI(kg/m2),						
<25	Reference			Reference		
≥25	0.49	0.25-0.94	0.034	0.51	0.26-0.98	0.045
Pathologic T stage						
Non-muscle invasive (pT0/Ta/T1/CIS)	Reference			Reference		
Muscle invasive (pT2)	0.79	0.35-1.79	0.576	0.53	0.22-1.29	0.165
Extravesical (pT3/T4)	2.93	1.68-5.10	<0.001	1.91	0.98-3.71	0.057
Variant histology of UC						
Absent	Reference					
Present	0.64	0.27-1.48	0.294			
LVI						
Absent	Reference			Reference		
Present	2.41	1.44-4.05	0.001	1.94	1.03-3.65	0.039
Perivesical margin						
Absent	Reference			Reference		
Present	9.37	1.27-68.75	0.028	3.69	0.48-28.26	0.209
Extent of PLND						
Limited	Reference			References		
Standard	0.65	0.23-0.98	0.045	0.59	0.33-1.08	0.089
Extended	0.10	0.01-0.80	0.030	0.29	0.04-2.33	0.243
Number of LNs removed (continuous)	0.92	0.89-0.96	<0.001	0.91	0.88-0.95	<0.001
Adjuvant CTx.						
Not done	Reference			Reference		
Done	1.79	1.00-3.18	0.047	0.83	0.38-1.81	0.643

HR=hazard ratio; CI=confidence interval; BMI=body mass index; LVI=lymphovascular invasion; PLND=pelvic lymph node dissection; LNs=lymph nodes; CTx=chemotherapy
